# Supplementary material for: Heart Failure Impacts Endothelial Cell Responses to Cardiac Surgery on Cardiopulmonary Bypass
Source: Cells. 2025 Aug 31;14(17):1357. doi: 10.3390/cells14171357 (PMC12428422; doi:10.3390/cells14171357)
Supplement: Supplementary file 1 [file cells-14-01357-s001.zip › cells-3823125-supplementary.pdf]

SUPPLEMENTAL INFORMATION

Table S1. Intraoperative characteristics

	Heart failure (n=18)	Control (n=18)	*P* value
Procedure type			
Mitral valve plasty (%)	56	94	**0.018**
Tricuspid valve plasty (%)	33	50	0.500
Surgical left ventricular restoration (%)	39	0	**0.008**
Left ventricular assist device implantation (%)	22	0	0.104
Coronary artery bypass grafting (%)	39	6	**0.041**
Aortic valve replacement (%)	17	0	0.229
Aorta surgery (%)	11	6	1.000
Medication use			
Epinephrine (%)	28	0	**0.045**
Noradrenaline (%)	94	44	**0.003**
Dobutamine (%)	83	33	**0.006**
Milrinone (%)	94	17	**<0.001**
Procedure characteristics			
Hypothermia during CPB (°C) (IQR)	32 (32-37)	32 (30-33)	0.265
CPB time (min) (IQR)	174 (120-214)	143 (103-225)	0.696
Cross clamp time (min) (±SD)	135±37	132±52	0.854
Procedure time (min) (IQR)	300 (254-388)	243 (188-326)	0.064
Medication use			
Max dose Nor on ICU (µg/kg/min) (IQR)	0.18 (0.08-0.6)	0.03 (0-0.13)	0.002
Total duration Nor on ICU (min) (IQR)	1281 (215-1615)	177 (0-882)	0.009
Total Nor on ICU (µg/kg) (IQR)	186.51 (14.77-345.03)	3.61 (0-41.60)	0.003

Footnote: Data presented as mean ± SD, median with interquartile range (IQR), or percentage for count data. Fisher exact test was used for count data, independent Student t-test was used for data presented as mean ± SD, and Median Mann–Whitney U test was used for data presented as median with interquartile range (IQR).

Table S2: STROBE Statement

STROBE Statement—Checklist of items that should be included in reports of ***cohort studies***

	Item No	Recommendation	Page No
**Title and abstract**	1	(*a*) Indicate the study's design with a commonly used term in the title or the abstract<br>(*b*) Provide in the abstract an informative and balanced summary of what was done and what was found	1,4-5
**Introduction**			
Background/rationale	2	Explain the scientific background and rationale for the investigation being reported	6-7
Objectives	3	State specific objectives, including any prespecified hypotheses	7
**Methods**			
Study design	4	Present key elements of study design early in the paper	8
Setting	5	Describe the setting, locations, and relevant dates, including periods of recruitment, exposure, follow-up, and data collection	8
Participants	6	(*a*) Give the eligibility criteria, and the sources and methods of selection of participants. Describe methods of follow-up<br>(*b*) For matched studies, give matching criteria and number of exposed and unexposed	8

Variables	7	Clearly define all outcomes, exposures, predictors, potential confounders, and effect modifiers. Give diagnostic criteria, if applicable	7-8
Data sources/ measurement	8*	For each variable of interest, give sources of data and details of methods of assessment (measurement). Describe comparability of assessment methods if there is more than one group	8-12
Bias	9	Describe any efforts to address potential sources of bias	NA
Study size	10	Explain how the study size was arrived at	8
Quantitative variables	11	Explain how quantitative variables were handled in the analyses. If applicable, describe which groupings were chosen and why	12
Statistical methods	12	(*a*) Describe all statistical methods, including those used to control for confounding<br>(*b*) Describe any methods used to examine subgroups and interactions<br>(*c*) Explain how missing data were addressed<br>(*d*) If applicable, explain how loss to follow-up was addressed<br>(*e*) Describe any sensitivity analyses	12-13
**Results**			
Participants	13*	(a) Report numbers of individuals at each stage of study—e.g., numbers potentially eligible, examined for eligibility, confirmed eligible, included in the study, completing follow-up, and analyzed<br>(b) Give reasons for non-participation at each stage<br>(c) Consider use of a flow diagram	14

Descriptive data	14*	(a) Give characteristics of study participants (e.g., demographic, clinical, and social) and information on exposures and potential confounders<br>(b) Indicate number of participants with missing data for each variable of interest<br>(c) Summarize follow-up time (e.g., average and total amount)	14
Outcome data	15*	Report numbers of outcome events or summary measures over time	14-18
